# Vascular Disruption Therapy as a New Strategy for Cancer Treatment

**DOI:** 10.3390/ijms262010085

**Published:** 2025-10-16

**Authors:** Jesús Gómez-Escudero, Patricia Berlana-Galán, Elena Guerra-Paes, Irene Torre-Cea, Laura Marcos-Zazo, Iván Carrera-Aguado, Daniel Cáceres-Calle, Fernando Sánchez-Juanes, José M. Muñoz-Félix

**Affiliations:** 1 Departamento de Bioquímica y Biología Molecular, Universidad de Salamanca, 37007 Salamanca, Spain; jesuschgoes@usal.es (J.G.-E.); berlanagalan@usal.es (P.B.-G.); elegpaes98@usal.es (E.G.-P.); irenedltorrecea@usal.es (I.T.-C.); lauramarcos@usal.es (L.M.-Z.); icasdp@usal.es (I.C.-A.); danicc@usal.es (D.C.-C.); 2 Instituto de Investigación Biomédica de Salamanca (IBSAL), 37007 Salamanca, Spain

**Keywords:** vascular disruption, angiogenesis, tumor vasculature, vascular disruption agents

## Abstract

A functional blood vessel network is required to deliver oxygen and nutrients to the cancer cells for their growth. Angiogenesis, the formation of new blood vessels from pre-existing ones, is one of the major mechanisms to create this vascular network. Anti-angiogenic therapy was conceived as the inhibition of the cellular and molecular players involved in tumor angiogenesis such as vascular endothelial growth factor and its main receptors. Due to limitations of this therapy, different approaches of vessel modulation such as vascular normalization or vascular promotion have been studied showing benefits in different tumor models and clinical trials. In contrast to anti-angiogenic therapy, which inhibits the blood vessels that are being formed, vascular disruption therapy aims to destroy already formed tumor vessels. These malignant vascular structures differ from other blood vessels in terms of endothelial cell states, pericyte coverage and basement membrane development. The molecules used for vascular disruption are microtubule-binding molecules, flavonoids that induce endothelial cell apoptosis or molecules vectorized to endothelial receptors. Many vascular disruption agents have been tested in clinical trials showing some promising results, but with some limitations that include resistant rim cells or the development of hypoxia that induces cancer regrowth and poor delivery of the anti-tumor agents. The main objective of this review is to focus on vascular disruption agents therapy, novel molecules, new ways to overcome therapy resistance to them, current clinical status and, especially, the upcoming challenges and applications of these molecules.

## 1. Introduction

Solid tumors are composed of masses of malignant cells that receive their supplies from blood. From early stages of cancer research, in 1971, a hypothesis presented that small, avascular tumors (1–2 mm^3^) cannot continue to grow until they develop their own blood vessel system [1]. Although, through the years, it has been discovered that cancer cells are able to survive by displacing non-tumoral cells that surround the already established blood vessels, taking the benefit of oxygen and nutrients supply without the necessity of the formation of new blood vessels, targeting the growth of new vascular structures continues to be one of the therapy approaches in cancer [2,3,4]. This line of treatment, called anti-angiogenic therapy (AAT), aims to impair the formation of new vessels. Although at first it showed some favorable results in clinic, the appearance of some resistant mechanisms made researchers move forward, looking for other approaches to target the vascular system, such as the use of vascular disruption agents (VDAs). VDAs are molecules that can cause internal tumor death by disrupting the immature tumor blood vessels [5]. These treatments have the advantage over other types of therapy because they do not need to diffuse across the whole tumor mass to reach the malignant cells. Thus, the mechanism of action of VDAs impairs the supply of nutrients for the tumor growth through a rapid shutdown of the tumor vasculature, leading to an increase in vascular permeability and extensive ischemic necrosis, with the consequence being the inhibition of the growth of tumors [6]. VDAs’ line of action varies between molecules that induce tumor necrosis following administration due to sudden vascular collapse [7,8] to others that cause vessel occlusion [9]. VDAs’ clinical relevance, due to their ability to selectively target tumor blood vessels to block blood flow, was enhanced particularly in cases of resistant neoplasms or those with higher malignancy grades [10]. However, the limitations of VDAs in clinics have been highlighted, including the generation of tumor hypoxia and off-target toxicities, the most notable being an associated cardiotoxicity [5]. In this review, we describe the diverse approaches to target tumor blood vessels to further focus on the different VDAs, their line of action, their related acquired-resistance mechanisms and the strategies to overcome them. We conclude with an update on the current status in clinical practice and the future perspectives of this promising therapeutic approach in cancer.

## 2. Vascular Development

### 2.1. Main Mechanisms of Tumor Vascularization

In physiological conditions, the vascular network plays an important role in maintaining tissue homeostasis. Its principal function consists of providing a continuous and efficient delivery of oxygen and nutrients to the cells, as well as removing waste metabolites [11]. Moreover, an adequate vasculature system contributes to the regulation of blood pressure, corporal temperature and to the transport of immune cells and signaling molecules that coordinate defense and tissue damage repair [12].

**Figure 1:**
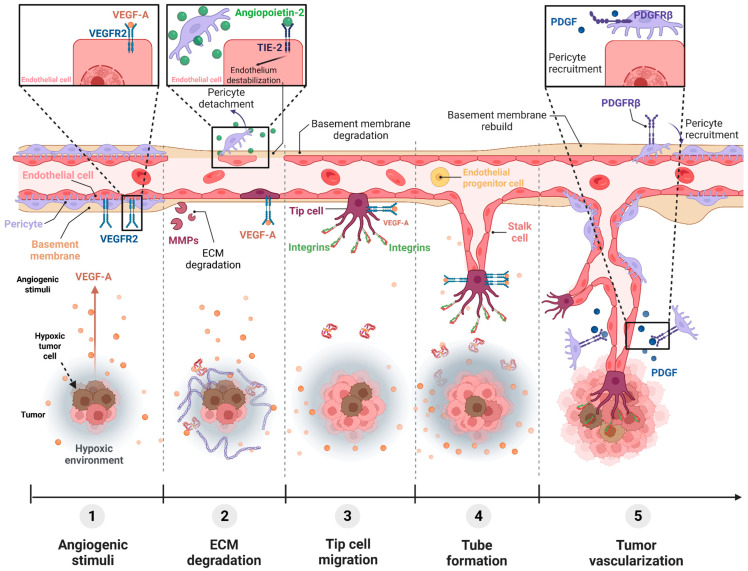
Molecular pathways involved in the angiogenic process during tumor growth. (1) Under hypoxic conditions, cancer cells secrete the VEGF, which activates its receptor VEGFR2. (2) Matrix metalloproteinases (MMPs) degrade basement membrane from the pre-existing blood vessel and the Angiopoietin-2 (Ang-2)-Tie pathway promotes pericyte detachment. (3) Under angiogenic stimulation, the tip cell migrates and (4) the stalk cell proliferates, forming sprouts. (5) PDGF secreted by ECs promotes pericyte recruitment and vessel stabilization. Figure created using https://BioRender.com/jqkksa6 accessed on 3 October 2025. BioRender.com (License: YL28TUYP4M).

The main blood vessel formation mechanism is the sprouting angiogenic process, which consists of the generation of new vessels from pre-existing ones [2]. This mechanism is strictly regulated by the angiogenic switch, which depends on the balance between pro-angiogenic and anti-angiogenic molecular factors. Under basal conditions, the endothelium remains quiescent due to the predominance of anti-angiogenic factors, whereas during embryonic development or wound healing, pro-angiogenic factors are secreted and trigger blood vessels formation (Figure 1) [3,4,13].

The angiogenic switch may also occur in pathological conditions such as cancer. Tumor cells rely on an extremely active metabolism to maintain a high rate of cell proliferation. Thus, they need a constant supply of oxygen and nutrients from the vascular system [14,15]. In the early stages of cancer development, tumors grow without forming new blood vessels and absorb nutrients from the surrounding extracellular space via diffusion [16]. However, once tumor size exceeds 2 mm^3^, diffusion uptake of nutrients is not sufficient to maintain tumor growth and cancer cells undergo hypoxia [1]. Under such conditions, the hypoxia-inducible factor 1α (HIF-1α) translocates to the nucleus and binds to hypoxia response elements (HREs) in the DNA, activating the expression and secretion of pro-angiogenic molecules, with the vascular endothelial growth factor (VEGF) being the main one [16]. This cytokine binds to its receptor vascular endothelial growth factor receptor 2 (VEGFR-2) and triggers intracellular signaling pathways such as PI3K/AKT, MEK/ERK or MAPK, which stimulate endothelial cell (EC) proliferation, migration and survival [14,16,17,18,19]. As a result, new vascular branches are formed and infiltrate into the tumor, supporting its growth [14].

Although sprouting angiogenesis is the main mechanism for tumor vascularization, others have been described in the past years. One of them is intussusception or non-sprouting angiogenesis, firstly characterized by Burri & Taret [20]. In this process, a pre-existing blood vessel is divided into two through the reorganization of the intercellular junctions between ECs, so no cell proliferation is required [16,21]. The advantage of this mechanism is that it is faster than sprouting angiogenesis and less metabolically demanding, allowing tumors to adapt rapidly to changes in the tumor microenvironment (TME). Moreover, blood vessels formed by this process show physiological levels of permeability, which ensures a correct blood flow to tumors [21]. On the other hand, some tumors develop their vasculature through vasculogenesis, the formation of new blood vessels from bone-marrow-derived ECs precursors called angioblasts [16,21,22]. These cells are recruited in the TME and produce tubular structures that give rise to blood vessels [22,23]. A less common mechanism, limited to certain specific tumor types, is vascular mimicry, which is highly dependent on hypoxia. Under such conditions, tumor cells undergo epithelial-to-mesenchymal transition (EMT) and migrate to form vessel-like structures capable of integrating with the pre-existing vasculature, thereby facilitating access to the blood supply [16,23]. Additionally, other tumors vascularize through the trans-differentiation of cancer stem cells into ECs and pericytes, thus generating new blood vessels [23]. Finally, one of the most characterized processes for tumor vascularization is vessel co-option (VCO), in which cancer cells hijack pre-existing blood vessels instead of generating new ones [24].

### 2.2. Normal Blood Vessels vs. Tumor Blood Vessels Characteristics

**Figure 2:**
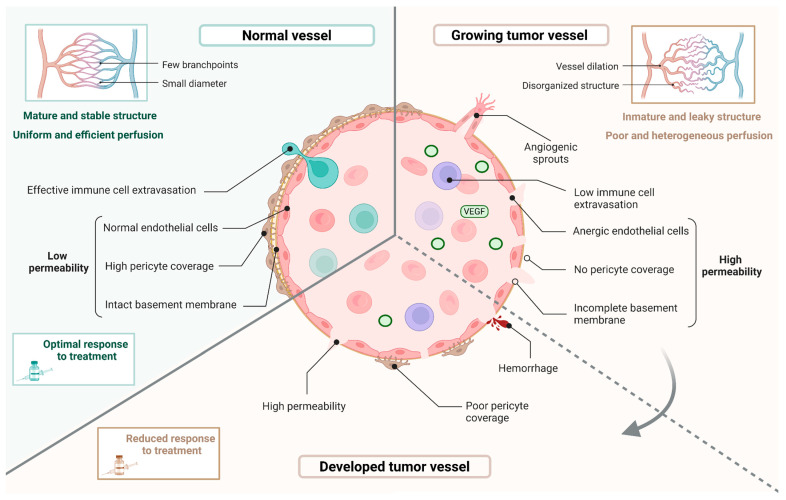
Features of normal vasculature versus the development of tumor vessels. Normal blood vessels exhibit a mature endothelial wall, with quiescent, tightly connected endothelial cells supported by an intact basement membrane and covered by pericytes. This structure ensures low permeability, selective molecular transport and effective extravasation of immune cells such as T CD8^+^ lymphocytes. The vascular network is composed of vessels with homogeneous diameters adapted to the tissue architecture and forming anastomoses, resulting in uniform and efficient perfusion and contributing to better therapeutic responses. In contrast, tumor vessels undergo distinct stages of development. During angiogenesis, the endothelial wall is immature, with endothelial cells at various differentiation stages, exhibiting loose junctions, a discontinuous basement membrane and minimal pericyte coverage. These vessels may form VEGF-induced sprouts and display high non-specific permeability, impairing immune cell infiltration. In the established tumor vasculature, these features largely persist, with no increase in pericyte coverage and reduced sprouting due to lower VEGF levels. Overall, the tumor vascular network is disorganized, with heterogeneous vessel diameters and poor perfusion, leading to reduced efficacy of intravenous therapies and promoting tumor progression and metastasis. Scheme created with https://BioRender.com/u4x1cow accessed on 3rd October 2025. Biorender.com (License: UL28TUYUY7).

Tumor blood vessels differ significantly from normal vasculature, impacting tumor growth, metastasis and therapeutic response [18]. In healthy tissues, the vascular network follows a well-defined organization, composed of large arteries that branch into smaller arterioles and capillaries, before converging into venules and veins [15,25]. This hierarchical architecture ensures uniform and efficient perfusion, allowing the delivery of oxygen and nutrients and adequate immune cell infiltration (Figure 1). By contrast, tumor blood vessels show a highly disorganized and abnormal morphology as a consequence of the fast proliferation of cancer cells that exert compressive strength on the vascular walls. These vessels are tortuous and have irregular diameters, with widened areas or constricted without a visible lumen [15,18,23,26,27]. Furthermore, tumor vasculature has excessive and irregular branching patterns, with numerous isolated bifurcations that form a disconnected vascular network [26]. Previous studies have shown micrography images of both normal and tumor vessels, in which the aberrant structure of tumor vessels can be appreciated in contrast to the well-organized structure of normal vessels [28,29]. In addition, as blood vessel formation during cancer is poorly regulated and lacks proper control mechanisms, they exhibit an immature structure characterized by a discontinuous basal membrane, defective EC-EC junctions and partial pericyte coverage [15,27] (Figure 2). Consequently, all these structural abnormalities are responsible for high permeability, vascular leakage and reduced blood flow, resulting in hypoxia that stimulates tumor progression and metastasis [30,31,32,33]. Permeability quantification assays performed in the past years have shown that tumor vessels exhibit increased permeability compared to normal vessels, on the order of 10^−7^ cm/s versus 10^−8^ cm/s, respectively [34,35,36]. Of particular concern is that this aberrant tumor vasculature significantly comprises the efficacy of anti-tumoral therapies, such as chemotherapy. Firstly, the structural and functional abnormalities hinder the adequate delivery of the therapeutic agents due to the high interstitial pressure within the tumor [30,37]. Additionally, the hypoxia-driven unfavorable TME may reduce drug activity and acquired resistance to treatment arises [15].

## 3. Strategies to Target Tumor Vasculature

**Figure 3:**
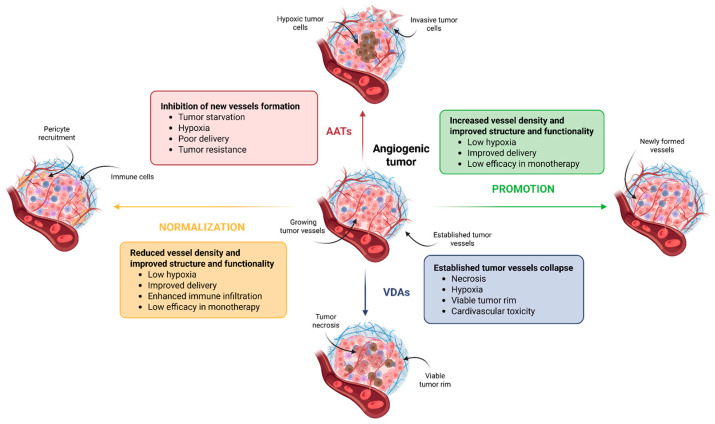
Major therapeutic approaches to target tumor vasculature: anti-angiogenic therapy, vascular promotion, vascular disruption agents and vascular normalization. AAT targets the angiogenic process by inhibiting the growth of newly developing vessels that supply nutrients to the tumor. As a consequence, high levels of hypoxia are generated, limiting the distribution of potential combination therapies. Increased hypoxia is associated with enhanced tumor malignancy, driving cancer cells toward a more invasive phenotype. Vascular promotion strategies aim to stimulate the formation of new vessels in order to improve drug distribution and reduce hypoxia. This approach is particularly useful in combination with conventional therapies such as chemotherapy and immunotherapy. VDAs act on already established tumor vessels. The vascular collapse they induce promotes tumor cell necrosis but also increases hypoxia. Resistance may emerge in the peripheral regions of the tumor, described as the viable tumor rim. Owing to their mechanism of action, VDAs are frequently associated with significant cardiovascular toxicity. Vascular normalization approaches seek to restore the normal structure and functionality of tumor vessels. In this process, the recruitment of pericytes plays a crucial role. Vessel restructuring improves blood perfusion, reduces hypoxia, and enhances immune cell infiltration, thereby facilitating the delivery of conventional therapies to tumor cells. Scheme created with https://BioRender.com/2blk8ua accessed on 3 October 2025. Biorender.com (License: PR28TUZ1R9).

In order to overcome the limitations caused by the abnormal architecture and structure of tumor blood vessels, targeting tumor vasculature has emerged as a promising approach in cancer therapy. Vessel modulation strategies can be divided into four groups: anti-angiogenesis, vascular normalization, vascular promotion and vascular disruption (Figure 3). Each approach targets different aspects of tumor blood vessels to inhibit tumor growth and improve anti-tumoral treatment outcomes.

### 3.1. Anti-Angiogenic Therapy

AAT was proposed in the early 1970s aiming to inhibit the formation of new blood vessels from pre-existing ones by blocking the angiogenesis process and depriving cancer cells of essential nutrients and oxygen [1]. This strategy was specifically designed to inhibit VEGF-signaling pathways [38]. Depending on their mechanism, AAT strategies can be divided into three groups. First, VEGF inhibitors prevent the binding of VEGF isoforms to their receptor, such as bevacizumab, the first AAT drug approved [38,39]. Second, there are monoclonal antibodies that block VEGF receptors directly. Finally, other inhibitors target the tyrosine kinase activity of VEGF receptors, sorafenib and sunitinib being the most common [38]. Although pre-clinical studies were promising, AAT administered in monotherapy has shown limited benefits [4,17]. The main hypothesis to explain these outcomes is that some tumors are unresponsive to AAT because they exploit alternative vascularization mechanisms [40,41]. Furthermore, angiogenesis can be mediated through VEGF-independent pathways [38]. Moreover, as AAT reduces tumor blood vessels, oxygen supply to tumors is limited, resulting in hypoxia that increases cancer aggressiveness and stimulates tumor cells’ migration and metastasis (Table 1) [3]. Finally, AAT has shown important secondary adverse effects, such as hypertension, proteinuria, endocrine disturbances or cardiac damage, among others [38,42]. AAT has only been effective when administered in combination with chemotherapy. Phase III clinical trials combining bevacizumab with chemotherapy have demonstrated improvements in progression free-survival and overall survival in metastatic colorectal cancer and non-small cell lung cancer (NSCLC) [43,44].

### 3.2. Vascular Normalization

In 2001, Rakesh K. Jain proposed the concept of vascular normalization, inspired by the paradoxical outcomes observed from the combination of AAT and chemotherapy [19]. Intuitively, the impairing of tumor vasculature by AAT should hinder chemotherapy delivery, resulting in treatment failure and worsening tumor progression. Nevertheless, pre-clinical data revealed enhanced responses when AAT was co-administered with chemotherapy [43,44]. For that reason, this author hypothesized that AAT may prune the excess of tumor angiogenic blood vessels and restore the aberrant and disorganized architecture of the remaining vessels into a physiologically functional vascular network with similar characteristics to normal blood vessels, including tighter ECs junctions, higher pericyte coverage, reduced tortuosity or enlarged diameter [19,37]. This process specifically targets immature tumor vessels formed by angiogenesis, thereby helping to restore the balance between pro-angiogenic and anti-angiogenic factors [4,45].

On the other hand, vascular normalization contributes to the establishment of a favorable TME. Initially, the aberrant architecture of tumor vessels leads to hypoxic conditions that impair the proliferation and function of effector immune cells and promote the activity of immunosuppressive cells such as regulatory T cells (Tregs) or M2 tumor-associated macrophages (TAMs). However, vascular normalization favors the infiltration of effector immune cells (M1 TAMs, T CD8^+^ cells, T CD4^+^ cells or natural killer cells) into the tumor (Table 1) [46,47]. For that reason, in recent years AAT has been combined with immunotherapy in clinical trials with favorable outcomes in NSCLC, gastric cancer and urothelial carcinomas [48,49]. In fact, several combined treatments of AAT and immunotherapy are approved in clinical practice for the treatment of different types of tumors [50]. Examples of anti-angiogenic agents used for vascular normalization include bevacizumab, axitinib or cabozantinib, among others [45]. In addition to these, several studies have demonstrated the potential therapeutic efficacy of anti-VEGF and anti-Angiopoietin-2 molecules [51,52,53]. It has been shown that VEGF inhibition transiently normalizes tumor vessels, whereas angiopoietin inhibition leads to a stable normalization of tumor vasculature, enhancing the anti-tumor immune response in both cases [51,52]. In view of this, antibodies that simultaneously block both the VEGF and Angiopoietin-2 have been employed for achieving vascular normalization [51,52].

Despite its therapeutic potential, vascular normalization remains a challenging strategy to implement clinically. Its efficacy varies depending on tumor type, individual patient characteristics or the specific AAT [30,32]. Moreover, the normalization effect is strongly dependent on the dose. In fact, lower doses have been shown to produce more favorable outcomes, whereas high doses may lead to vascular regression that ultimately counteracts the benefits of normalization [30,32,45,46]. In addition, this strategy is only effective within a narrow therapeutic “normalization window” [4,37,46]. Additionally, it has been proposed that vascular normalization only takes place in tumor regions where the imbalance between pro-angiogenic and anti-angiogenic molecules has been corrected [4].

### 3.3. Vascular Promotion

Vascular promotion is a therapeutic approach proposed by Wong et al. in 2015 consisting in increasing tumor blood vessel density and functionality in order to improve blood flow and enhance the delivery of anti-tumoral drugs so as to boost their effectiveness [3,54]. Therefore, opposite to vascular normalization, which maintains or even reduces the number of vessels while improving their quality, vascular promotion increases both vessel density and functionality (Table 1) [3]. These authors took advantage of the studies given a few years before by Reynolds et al., which demonstrated a pro-angiogenic effect of cilengitide at low doses, a RGD-mimetic α_v_β_3_ and α_v_β_5_ integrins inhibitor [55]. Given this information, they used the triple combination of the DNA synthesis blocker gemcitabine; verapamil, a calcium channel blocker that acts as vasodilator; and low doses of cilengitide in Lewis Lung Carcinoma (LLC) subcutaneous tumors and Pancreatic Ductal Adenocarcinoma (PDAC) models and observed a significant tumor growth reduction [54]. In addition to this work, other vascular promotion strategies have been proposed by other groups, such as the use of LPA treatment, which promotes a fine vascular network in LLC tumor, and the utilization of the tubulin-binding drug eribulin mesylate, which increases tumor blood vessels and perfusion in xenograft models [56,57]. Vascular promotion strategies can be interesting in hypovascular and desmoplastic solid tumors, as suggested by Wong et al., 2016 [3]. There are several aspects that need to be considered to set up vascular promotion therapies. First is to properly design the schedule of the treatments, remembering that the promotion of angiogenesis in combination with the limited effects of chemotherapies or immunotherapies can increase tumor growth.

### 3.4. Vascular Disruption

**Figure 4:**
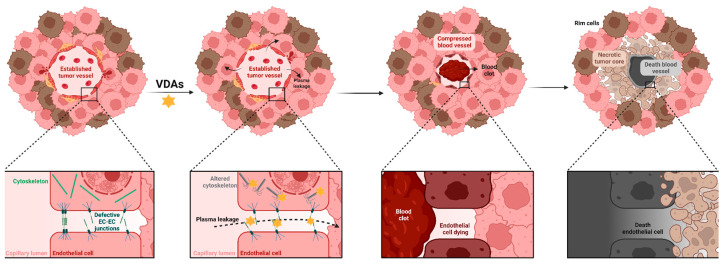
General mechanism of action of VDAs. VDAs are designed to target already-established tumor blood vessels, which are immature and display an abnormal architecture. When VDAs are administered, they alter cytoskeleton and endothelial cell junctions, which results in increased vessel permeability and interstitial pressure. Consequently, blood vessels are compressed, which favors clots formation and ultimately, vessels collapse and are destroyed. Moreover, depriving tumor cells of nutrients and oxygen induces necrosis in the core of the tumor, but not in the periphery, where a rim of cells remains and may give rise to rebounds. Golden star means VDA. Created with https://BioRender.com/2eq298q accessed on 3 October 2025. Biorender.com (License: CP28TUZ7OV).

Vascular disruption is a therapeutic strategy designed to selectively target the established vasculature of tumors, causing rapid vessel collapse and tumor necrosis in 1–6 h after the treatment. This approach is based on the evidence that normal vasculature is both functionally and morphologically distinct from already developed tumor vessels [58,59]. These tumor-associated blood vessels represent a promising therapeutic target, as they are typically more immature and unstable than normal vasculature. This is largely due to an incomplete or absent extracellular matrix and poor pericyte coverage, resulting in high vascular permeability and a propensity for leakage and hemorrhage [60,61]. The action of VDAs involves profound alterations in the cytoskeleton and intercellular junctions of ECs, increasing vessel permeability, leading to plasma leakage, which results in elevated interstitial pressure that narrows vessel caliber and restricts blood flow. Furthermore, plasma extravasation heightens blood viscosity, promoting erythrocyte aggregation and thrombus formation that obstruct the vessel, which finally collapses [62]. Apart from affecting ECs, vascular disruption also destroys tumor cells by impeding the oxygen and nutrients supply, producing a necrotic area specifically in the tumor core but not in the periphery since these cells rely on pre-existing blood vessels, which are insensitive to VDAs [5,6,58,63] (Figure 4). Cytotoxic effects and apoptosis have also been reported in tumor cells, particularly in microtubule-binding agents, through activation of the caspase and JNK stress-response pathways [64,65]. On the other hand, by obliterating the vascular network, vascular disruption impedes the metastatic dissemination of tumor cells [5].

Unlike AAT treatment, which requires a prolonged dosing regime, the rapid action of VDAs allows their administration in intermittent dosing schedules, as prolonged drug exposure is not required to achieve vascular collapse [58,63]. Nevertheless, vascular recovery has been observed within 24 h, highlighting the need for frequent dosing or combination with other therapies to achieve sustained anti-tumor effects [66].

Despite their mechanistic appeal, VDAs face several limitations. A consistent observation in pre-clinical and clinical studies is the persistence of a viable rim of tumor cells at the periphery, which may lead to regrowth [5,66]. In addition, the hypoxic environment induced by VDAs may paradoxically promote tumor migration and aggressiveness [5,67,68]. VDAs are associated with cardiovascular toxicity, being the most frequent acute hemodynamic changes shortly after drug administration. These effects are typically transient and manageable with standard cardiac medications and blood pressure monitoring [68]. In recent years, nanoparticles designed for vascular disruption have been a novel advancing area for cancer treatment, aiming to overcome the limitations of standard VDAs such as off-target toxicity and tumor recurrence. Thus, nanoparticles are designed to improve targeting, combining multiple therapeutic modalities and enabling real-time monitoring. The strategies combine the use of several triggers such as light, ultrasound, ROS or the use of special polymers to provide a dual drug release, or the utilization of photothermal agents and photodynamic therapies which promotes synergetic effects to target tumors and prevent their regrowth.

Approaches comprise the use of enzymatically transformable polymer-based nanotherapeutic exploits matrix metalloproteinase (MMP) overactivation guiding the codelivery of colchicine and marimastat (an MMP inhibitor) [69], the combination of TNFa with gold nanoparticles [70], the use of nano-hydrogels [71] or an erythrocyte membrane-modified biomimetic synergistic nanosystem [72].

The advantage of the nanoparticles therapy approach is reflected by the enhanced drug penetration that overcomes the barriers imposed by the tumor microenvironment, the success on tumor suppression and metastasis inhibition or the possibility of real-time monitoring [73,74]. In addition, it may reduce both the toxicity associated with other strategies and also inflammation [69,75]. As it has been shown, pre-clinical results are encouraging, but further research is still needed to translate the approach for clinical use.

Moreover, it has been described that the combination of the VDA fosbretabulin (CA4P), iRGD peptide and polymersomes in the treatment of peritoneal carcinomatosis produced a threefold increase in nanoparticle accumulation and a more homogeneous distribution within tumor tissue. While CA4P enhanced vascular permeability, effective penetration of nanoparticles required iRGD, highlighting the value of sequential targeting for improved drug delivery in peritoneal carcinomatosis tumors expressing αv integrins and NRP-1. In survival studies, monotherapies were ineffective, but iRGD-based combinations extended survival by ~5 days; notably, the triple therapy also reduced tumor weight and uniquely decreased tumor nodules. Overall, CA4P pretreatment enhances the efficacy of iRGD-potentiated nanoparticle therapy, offering a promising and safe strategy to improve drug delivery in solid tumors [76].

In contrast to the mentioned vascular modulation strategies such as anti-angiogenesis, vascular normalization or vascular promotion that could consider that all blood vessels in a tumor are in the same development stage or with the same angiogenic requirements, the exploitation of VDAs in combination with antiangiogenic molecules offers a very interesting approach that considers that not all the blood vessels in a tumor are in the same development stage and show different levels of growth factor receptors, pericyte coverage or basement membrane composition.

## 4. Vascular Disrupting Agents

VDAs can be classified into two main categories: small molecule VDAs and ligand-directed VDAs. In turn, small molecules have been divided into two classes: microtubule-destabilizing agents and flavonoids, which induce tumor necrosis following administration due to sudden vascular collapse [7,8]. On the other hand, ligand-direct VDAs act directly on the tumor endothelium, causing vessel occlusion [9].

### 4.1. Microtubule-Destabilizing Agents

Microtubule-destabilizing agents interfere with ECs’ microtubules by binding to the colchicine domain of tubulin, resulting in vascular collapse and subsequent tumor necrosis [77]. Based on their chemical characteristics, microtubule-destabilizing agents can be subclassified into combretastatins, colchicines, phenylhistidines and synthetic compounds [9].

Among the combretastatins, fosbretabulin (CA4P) is notable for its ability to inhibit tubulin aggregation and EC proliferation. In addition, it enhances tumor sensitivity to chemotherapy and immunotherapy [78]. However, its limited specificity and high cardiovascular toxicity have restricted its clinical application (Table 2) [79]. Thus, although CA4P has advanced through multiple phase II trials in combination with chemotherapy and immunotherapy (NCT00113438/NCT00653939/NCT02279602/NCT02055690), it has not succeeded in phase III studies (NCT00507429). Other combretastatins, such as OXi4503 (CA1P) and AVE8062 (AC7700), have completed phase I and II clinical trials in solid tumors, respectively (NCT00977210/NCT01263886) [62,80,81].

Plinabulin (NPI-2358), a synthetic phenylhistidine analog, has demonstrated direct cytotoxic effects on tumor cells by inducing apoptosis, in addition to its vascular-disrupting activity [65]. This agent has successfully advanced to phase III trials in combination with docetaxel in patients with NSCLC and multiple myeloma (NCT02504489) [87,88,89].

During the past decade, efforts have focused on developing synthetic analogs intended to increase its therapeutic efficacy and selectivity. Poly(l-glutamic acid)-combretastatin A4 conjugate [82], orally bioavailable compounds such as DX1002 [78] and cis-restricted triazole analogs [79] are some of the most relevant emerging strategies. Further studies have concentrated on optimizing drug delivery systems through quaternary ammonium salt microspheres [83] and oncolytic polymers [84], which facilitate the controlled release of the disrupting agents (Table 2).

### 4.2. Flavonoids

Flavonoids are regarded as exerting a triple mode of action: induce cytokine production, promote EC apoptosis and activate the immune response, leading to selective shutdown of tumor vasculature [9,62]. Vadimezan (DMXAA/ASA404), the most significant small-molecule flavonoid, triggers Tumor Necrosis Factor α (TNF-α) release, which results in tumor necrosis. Beyond its role in cytokine synthesis, it also demonstrates a direct anti-vascular effect through the induction of EC apoptosis [90]. It showed promising results in early trials but failed in phase III due to specificity limitations in drug targets, as well as a high toxicity rate (NCT00738387/NCT00662597) [91]. Cardiovascular toxicity, retinal impairment and apraxia have been reported as severe side effects of this molecule [85].

To overcome these limitations, novel drug delivery mechanisms such as P@NPPD have been developed. This platelet-mediated system triggers the coagulation cascade, thereby self-amplifying the release of DMXAA. In this way, the strategy aims to enhance drug delivery to tumor cells while simultaneously mitigating the adverse effects of disrupting therapy (Table 2) [85].

### 4.3. Ligand-Directed Agents

Ligand-directed agents’ strategy consists of the delivery of toxins or pro-coagulants directly to tumor vasculature, promoting vessel occlusion [5]. Fusion proteins, immunotoxins, antibodies linked to cytokines, liposomally encapsulated drugs and gene therapy approaches are included in this category [8].

These agents can promote thrombosis either directly or indirectly, through mechanisms that include EC death, immune-mediated responses against tumor vasculature or structural remodeling of blood vessels [8]. To improve the specificity of these strategies, multiple molecular targets have been explored over the years. Among the most studied are the Major Histocompatibility Complex (MHC) class II marker [92], which plays a key role in antigen presentation; endoglin [93,94], a Transforming Growth Factor β (TGF-β) co-receptor highly expressed on proliferating ECs within tumors; and Vascular Cell Adhesion Molecule 1 (VCAM-1) [95], an adhesion molecule involved in leukocyte–endothelium interactions.

However, some markers are not specific only to tumor vessels such as CD31, CD34, vWF or UEA-1. Thus, overexpressed or specific tumor vessel molecules are better options as vascular direct ligand disruption targets, such as VEGFR2, Endomucin, Endoglin, MCAM, CD276, PSMA, ESM1, Fibrilin2, Emilin2, Lox, Serpin1, Sox17 or CD40 [96,97,98]. Other promising targets are integrins αvb3 or αvb5, STING or the IGFBP7 pathway [99,100].

Integrin-targeting agents exploit the overexpression of αvβ3 and α5β1 integrins in tumor-associated vasculature. For instance, RGD-(KLAKLAK)_2_ constructs trigger mitochondrial damage and endothelial cell death, which in turn leads to vascular collapse and tumor necrosis [101,102].

In parallel, VEGF receptor-targeting agents leverage the critical role of VEGF signaling in angiogenesis. Among these, fusion proteins such as ABRaA-VEGF121 selectively bind VEGFR-2 on tumor endothelium, delivering potent cytotoxins that promote vascular destruction and tumor necrosis, while importantly sparing normal tissues [5,103].

Moreover, tissue factor-based strategies, such as tTF-EG3287, couple a truncated tissue factor to a tumor-homing polypeptide. Through this design, the construct localizes specifically to tumor vasculature, thereby inducing thrombosis and inhibiting tumor growth in pre-clinical models [104].

Although ligand-directed agents exhibit high selectivity, their clinical application remains limited by factors such as elevated production costs and a relatively narrow spectrum of tumor targets [9]. In an effort to overcome these drawbacks, recent studies have explored the use of nucleic acid-loaded lipid nanoparticles (LNPs). These systems can be engineered to deliver either small interfering RNA (siRNA) against Fas ligand or a stimulator of interferon genes, thereby aiming to potentiate the therapeutic efficacy of conventional vascular-disrupting agents while broadening their applicability (Table 2) [86].

## 5. Resistance to Vascular Disruption

While VDAs can induce a profound reduction in tumor mass by targeting and destroying the tumor vasculature, their use as monotherapy has shown limited clinical efficacy, largely due to a variety of contributing factors, often leading to incomplete tumor eradication and recurrence [105] or the occurrence of undesirable adverse effects [68].

### 5.1. Viable Tumor Rim

After VDA treatment, necrosis does not occur in the peripheral regions of the tumor mass, leaving viable cells that may cause relapse in the patient, thus creating a peripheral resistance because these cells are supported by normal tissue vasculature or vessels with high pericyte coverage, making them less sensitive to VDAs [105,106]. Another possibility is that those cells are able to survive due to the mechanism of VCO [11]. The most effective VDAs leave a thin ring of around 10% of viable cells [107]. This surviving rim is a major source of tumor regrowth, thus promoting recurrence and metastasis [107,108]. In addition to the running hypothesis that the ineffectiveness of VDAs to eradicate tumor rim cells is because these cells might nourish from surrounding normal vasculature, it is established that hypoxia-tolerant tumor rim cells are not ordinary tumor cells to target with conventional treatments [107].

### 5.2. Hypoxia-Induced Adaptation

VDAs induce hypoxia, eventually leading to central necrosis of the tumor mass [10]. This hypoxia pattern may select for tumor cell populations that are resistant to oxygen deprivation and exhibit more aggressive growth patterns [5,10]. This VDAs-induced hypoxia also upregulates genes involved in angiogenesis and drug resistance, promoting new vessel growth and adaptation to low oxygen conditions [109].

### 5.3. Cellular and Microenvironmental Factors

Different TME components of the tumors can contribute to the resistance against VDAs. It has been observed that vessels with high pericyte coverage are more resistant to VDAs [110]. Pericytes stabilize and promote the maturation of vessels, making them less susceptible to disruption leading to the survival of the tumor rim after VDAs treatment, as the pericyte coverage is more prominent at the tumor periphery [111]. Furthermore, pericytes induce survival signals in ECs, such as the upregulation of anti-apoptotic and autocrine VEGF-signaling pathways, protecting ECs from VDAs-induced cytotoxicity [112]. Targeting pericytes can help overcome this resistance [113]. Other cell types such as the TAMs and endothelial progenitor cells (EPCs) can contribute to vessel repair and regrowth after VDAs therapy [114]. TAMs can contribute to VDAs’ resistance because some of them, especially the TIE2-expressing macrophages (TEMs), promote angiogenesis, and these are increased by VDAs therapy [115,116].

### 5.4. Strategies to Overcome Vascular Disruption Resistance

Resistance to VDAs is a major barrier to their effectiveness in cancer therapy, often resulting in tumor regrowth from surviving peripheral cells. Several strategies have been developed to overcome this resistance, focusing on targeting the resistant tumor rim, blocking repair mechanisms and using combination therapies (Table 3). One of these approaches is targeting pericyte-covered vessels. For this purpose, the strategy is to develop prodrugs that specifically target pericytes, which protect peripheral tumor vessels from VDAs. One example of this is the FAPα-activated prodrug Z-GP-DAVLBH, that selectively destroys pericyte-rich vessels, eradicating the otherwise VDAs-resistant tumor rim and leading to complete tumor regression in pre-clinical models [110]. A second approach can be blocking EPC recruitment. This involves the combination of VDAs with agents that inhibit the late mobilization of circulating EPCs, which tumors use to repair damaged vasculature after VDAs treatment [117].

A combination of chemotherapy and immunotherapy is a promising strategy to overcome VDA resistance and to enhance the efficacy of these treatments. As mentioned in previous sections, VDAs often leave a viable rim of tumor cells at the periphery. To destroy these rim cells that are resistant to blood-flow starvation, the use of chemotherapy and immunotherapy can overcome this limitation to VDAs [58]. In addition, sequential administration may also be useful. Administration of VDAs with other conventional treatments represents the optimal strategy to enhance anti-tumor activity. Administration of VDAs should aim to minimize alterations in blood flow in order to maintain effective delivery of the chemotherapeutic agent [118]. Sequential or concurrent administration of VDAs with chemotherapy or radiotherapy targets both the tumor core and the resistant rim, improving overall tumor control [119]. Regarding combination therapies with immunotherapy, VDAs can enhance immune cell infiltration, and combining them with immune checkpoint inhibitors may further improve outcomes [119].

On the other hand, targeting molecules labeled with ^131^I has been used in combination with VDAs to overcome poor antibody penetration in large tumors and hypoxic tissue’s radiotherapy resistance [120]. For example, the combination of ^131^I-A5B7 and CA4P has shown to eliminate most tumors in colorectal xenograft models and to prolong antibody retention [121]. A phase I clinical trial in gastrointestinal adenocarcinoma patients showed a partial response in only 1 of 10 cases [122]. Tumor-specific uptake has been confirmed, but absorbed dose comparisons were not reported and dose-limiting myelosuppression occurred. A combination of CA4P and ^131^I-hypericin has emerged as a sequential dual-targeting strategy, using CA4P to induce tumor necrosis and ^131^I-hypericin to selectively accumulate in necrotic zones [123]. Pre-clinical studies in mice and rabbits have demonstrated high necrosis selectivity, prolonged retention (>9 days) and significant tumor growth inhibition [123,124]. This approach may overcome VDAs’ resistance and allows visualization of residual tumor cells through nuclear imaging due to hypericin’s strong necrosis affinity.

In contrast, the combination of VDAs with AAT prevents new vessel formation and further starves the tumor, addressing both existing and newly forming vasculature [125].

Due to the complexity of the resistance mechanisms and the different treatment schedules required for combination therapies, overcoming VDAs’ resistance requires multi-pronged strategies, including targeting pericyte-protected vessels, blocking repair mechanisms and using combination therapies. These approaches were promising in pre-clinical and early clinical studies aiming for more effective and lasting tumor control. Moreover, spatiotemporally targeted nanoparticles can co-deliver VDAs and cytotoxic drugs, overcoming hypoxia-induced therapeutic resistance and improving drug penetration into tumors [6].

## 6. Current Status of Clinical Trial Studies of Vascular Disruption Agents

Initially, pre-clinical studies demonstrated that VDAs inhibit tumor growth. Specifically, they exhibit anti-tumor activity in advanced and refractory human cancers [5]. In recent years, although pre-clinical trials have highlighted their therapeutic potential, they have also simultaneously revealed possible limitations and suggested new approaches to overcome them. As explained above, the main current limitations identified in clinical trials include off-target toxicities of VDAs, most notably cardiotoxicity [5]. These agents may also exhibit side effects commonly associated with conventional cytotoxic therapies. Nevertheless, a significant number of VDAs has successfully passed intermediate and late phases of clinical trials [126].

The monitoring of the aforementioned resistance mechanisms has been explored through magnetic resonance imaging markers and, in order to address these challenges, more advanced clinical trials have investigated therapeutic combinations to optimize the cytotoxic effects of VDAs. These combination therapies may involve anti-angiogenic agents, chemotherapy, radiotherapy or immunotherapy—some examples are presented in Table 4 [10,126].

Ongoing clinical trials evaluate commonly used VDAs, several of which are listed in Table 4. Beginning with CA4P, it has been shown to induce significant tumor necrosis as a single agent, in addition to enhancing the efficacy of combination therapy with an anti-VEGF antibody such as bevacizumab [127]. In phase I clinical trials, where the pharmacokinetics and tolerability of CA4P were evaluated, intravenous administration every three weeks was generally established (NCT00003768, NCT00395434). Years later, combination therapy studies emerged in which treatment efficacy was assessed. However, in trials classified as terminated, the reported efficacy did not improve the overall clinical condition at the time. In the case of the combination with everolimus, there was an incidence of patients reporting at least one adverse event according to NCI CTCAE v4.0 during the evaluation of the maximum tolerated dose, which led to the termination of the trial (NCT03014297). Similarly, in the case of the combination with pazopanib, it was associated with severe but reversible cardiac toxicity (NCT02055690) [128].

NPI-2358, on the other hand, exhibits immunostimulatory effects that are beneficial in combination with immunotherapies such as nivolumab and ipilimumab in lung cancer [129], or in combination with docetaxel in platinum-resistant lung cancer [130]. The early-phase trials with Pinabulin (NPI-2358) demonstrated, through pharmacokinetic analysis, an optimal 28-day treatment cycle (NCT00322608). Regarding combination trials, most of them showed representative safety and efficacy profiles that allowed the studies to be completed (NCT00630110, NCT03575793). Other trials may have been terminated prematurely due to the approval of immunotherapy as a first-line treatment for the type of lung cancer under investigation (NCT02846792).

Additionally, DMXAA has demonstrated good clinical tolerability across a broad dosage range, showing differences in its mechanism of action compared to other agents affecting tumor vasculature [131]. Phase I trials with Vadimezan (DMXAA) demonstrated clinical anti-tumor activity and a reduction in tumor blood flow with well-tolerated doses administered intravenously over 20 min every three weeks, for up to 12 cycles (NCT00863733), or through six weekly doses in patients with refractory tumor (NCT00856336) [131,132]. Other trials were discontinued due to observed toxicities, some of which resembled serotonin syndrome [132], or because they failed to pass safety and pharmacokinetic evaluations when assessing ASA404 in combination with fluvoxamine, a selective serotonin reuptake inhibitor and CYP1A2 inhibitor (NCT01299415 and NCT01240642). However, its combination with platinum-based drugs, although well tolerated, did not enhance their efficacy [133]. A similar case is observed with AVE8062 (Ombrabulin), where maximum treatment of 21 cycles every three or six weeks is indicated (NCT00968916, NCT01263886) and whose combination with chemotherapeutic agents such as paclitaxel and carboplatin is tolerable (NCT01293630, NCT01332656) but does not improve first-line therapies [134,135], although it continues to be tested in combination with other treatments such as docetaxel [136]. BNC105P has also been tested in clinical trials to determine the recommended dose as well as pharmacotoxic and pharmacokinetic effects [137], including its combination with immunotherapy (NCT01034631, NCT03454165, NCT03647839). Finally, the agent NGR-hTNF, which targets solid tumors, appears to be highly selective for tumor neoangiogenic vessels and can be safely combined with other anti-tumor therapies such as doxorubicin (NCT00484432, NCT00484341, NCT03804866). It has shown efficacy, for instance, in platinum-resistant lung cancer [138,139]. Specifically, low doses of NGR-hTNF appeared to exert direct global effects on tumor vascular permeability and blood flow. The reduction in vascularization was more pronounced in patients who received at least four treatment cycles, suggesting that the overall effect tends to occur during the later stages of therapy. An optimal administration dose of 0.8 μg/m^2^ was determined (NCT00419328, NCT00994097) [138].

As future perspectives, continued pharmacological evaluation of combination treatments is warranted, along with the identification of tumor biomarkers that can indicate when it is appropriate to administer VDAs. This could ultimately promote the development of effective personalized treatments while minimizing potential side effects [126].

## 7. Conclusions and Future Challenges

This literature review has highlighted the great therapeutic potential of VDAs in cancer treatment. As explained above, the future of VDAs lies in combination therapies with chemotherapy and immunotherapy, always taking into account the possibility of resistance developing. The complexity of tumor blood vessels, and especially their heterogeneity, requires prior knowledge of how these vessels work and possibly the simultaneous use of different vascular modulation agents.

The development of blood vessels demands different angiogenic requirements, varying levels of growth factors such as the VEGF, and diverse levels of their receptors such as VEGFR-2. This complexity determines the variety of responses to the vascular modulation therapies explained in this review, such as anti-angiogenesis, vascular normalization, vascular promotion or vascular disruption. The study of vascularization status in biopsies must be as accurate as possible in patients who are going to receive these molecules in combination with immunotherapies or chemotherapies. However, there is a major limitation due to the heterogeneity of the malignant area in terms of which area of the tumor is representative in the biopsy. In addition, there are tumors that cannot be accessed by biopsy. For all these reasons, the combination of several of these vascular modulation strategies must be taken into account. Given the resistance mechanisms to AAT, and the emergence of non-angiogenic vascularization processes such as VCO or vascular mimicry, the combination of AAT and VDAs can represent an opportunity to overcome these limitations.

The next challenge in the design of pre-clinical and clinical studies using VDAs is to know the molecular features of the blood vessels that sensitize them to VDA treatment and modify them to target more specifically malignant vessels compared to non-tumoral blood vessels or physiological angiogenic vessels. As we have mentioned in previous sections, necrosis caused by VDAs can lead to changes in the cytokines secreted by vascular cells or even cancer cells that modify tumor microenvironment, especially in the population of immunosuppressive tumor cells such as M2 macrophages. The modulation of these effects need to be studied further.

Moreover, deeper insight about the molecular mechanism and the in vivo validation of secondary adverse effects will be a priority to research.

## Figures and Tables

**Table 1:** Strategies to overcome tumor vasculature.

Strategies	Mechanism	Advantages	Disadvantages
Anti-angiogenic therapy (AAT)	Inhibition of new blood vessel formation	- Tumor starvation - High efficacy in combination with chemotherapy	- Widespread hypoxia - Tumor resistance - Poor drug delivery - Low efficacy in monotherapy - Adverse effects
Vascular normalization	Reduced vessel density and improved structure and functionality	- Low hypoxia - Improved drug delivery - Enhanced immune cells infiltration	- Narrow therapeutic window - Low efficacy in monotherapy
Vascular promotion	Increased vessel density and improved structure and functionality	- Low hypoxia - Improved drug delivery - Enhanced immune cells infiltration - Broad therapeutic window	- Low efficacy in monotherapy - Possible enhanced tumor growth
Vascular disruption	Collapse of established angiogenic vessels	- Tumor necrosis - Rapid effect - Local hypoxia - Effective against AAT resistance	- Tumor rim cells resistance - Cardiovascular toxicity - Low efficacy in monotherapy

**Table 2:** VDAs’ key features and limitations.

VDAs	Compounds	Mechanism	Features	Enhancing Strategies	References
Microtubule-binding	CA4P, OXi4503	Endothelial cytoskeleton collapse	- Low specificity - High toxicity - Low cost - Poor delivery	Improved-selectivity analogs	Liu et al., 2017 [82] Wei et al., 2025 [78] Prieto et al., 2025 [79]
				Improved delivery systems	Su et al., 2025 [83] Li et al., 2025 [84]
Flavonoids	DMXAA	Cytokine induction, apoptosis	- Low specificity - High toxicity - Low cost - Poor delivery	Cell-mediated delivery systems	Chu et al., 2025 [85]
Ligand-directed	Antibodies, peptides	Targeted toxin/pro-coagulant delivery	- High specificity - Low toxicity - High cost - Target limitation	Lipid nanoparticle-mediated ligand silencing	Endo et al., 2025 [86]

**Table 3:** Strategies to overcome VDA resistance.

Strategy	Mechanism of Action	Compound	Reported Benefits
Pericyte targeting	Selective ablation of pericyte-covered vessels	Z-GP-DAVLBH	Elimination of VDA-resistant tumor rim
EPC inhibition	Blockage of post-treatment vascular repair	TKR inhibitors + CA4P/VEGFR2 inhibitors + CA4P	Reduced neovascularization
Chemotherapy combination	Cytotoxic effect on surviving peripheral tumor cells	Conventional therapies (Carboplatin, Cisplatin, etc.) + VDAs	Enhanced tumor regression, including viable rim
Immunotherapy combination	Increased immune infiltration and activation	Immune check-point inhibitors + VDAs	Improved immune-mediated tumor clearance
Radiotherapy combination	Selective radiation of necrotic and hypoxic tumor regions	^131^I-A5B7 + CA4P/^131^I-hypericin	Tumor necrosis targeting, imaging capability
Anti-angiogenic therapy	Inhibition of neovascularization	VDAs + AAT	Dual targeting of existing and forming vasculature
Nanoparticle co-delivery	Coordinated delivery of VDAs and cytotoxins	VDAs + drug-loaded nanoparticles	Improved penetration, reduced hypoxia-driven resistance

**Table 4:** Representative clinical trials in the area of vascular disruption in cancer.

Clinical Trial Code	Treatment (Drug)	VDA Target	Pathology Conditions	Start Year, Phase and Status
NCT00003768	Fosbretabulin (CA4P)	Tubulin	Unspecified Adult Solid Resistant Tumor	1998 Phase I Completed
NCT00113438	Fosbretabulin (CA4P) Paclitaxel Carboplatin	Tubulin	Advanced Imageable Malignancies	2005 Phase II Completed
NCT00395434	Fosbretabulin (CA4P) Bevacizumab (Avastin)	Tubulin VEGF	Advanced solid tumors	2006 Phase I Completed
NCT00507429	Fosbretabulin (CA4P) Carboplatin Paclitaxel	Tubulin	Anaplastic thyroid carcinoma	2007 Phase II, III Terminated
NCT00653939	Fosbretabulin (CA4P) Carboplatin Paclitaxel Bevacizumab	Tubulin VEGF	NSCLC Lung Cancer	2008 Phase II Completed
NCT02279602	Fosbretabulin (CA4P)	Tubulin	Neuroendocrine Tumors	2014 Phase II Completed
NCT02055690	Fosbretabulin (CA4P) Pazopanib	Tubulin	Advanced Recurrent Ovarian Cancer	2014 Phase Ib/II Terminated
NCT03014297	Everolimus Fosbretabulin (CA4P)	Tubulin	Neuroendocrine Tumors	2017 Phase I Terminated
NCT00322608	Plinabulin (NPI-2358)	Tubulin	Advanced Solid Tumor Malignancies Lymphoma	2006 Phase I Completed
NCT00630110	Plinabulin (NPI-2358) Docetaxel	Tubulin	Advanced NSCLC	2008 Phase I, II Completed
NCT02812667	Plinabulin (NPI-2358) Nivolumab	Tubulin	NSCLC Metastatic	2016 Phase I Active, not recruiting
NCT02846792	Plinabulin (NPI-2358) Nivolumab	Tubulin	Stage IIIB-IV NSCLC	2017 Phase I, II Terminated
NCT03575793	Plinabulin (NPI-2358) Nivolumab Ipilimumab	Tubulin	Recurrent Small Cell Lung Cancer	2018 Phase I, II Completed
NCT05130827	Plinabulin (NPI-2358) Pegfilgrastim	Tubulin	Multiple Myeloma	2021 Phase II Active, not recruiting
NCT00863733	Vadimezan (DMXAA/ASA404)	Pro-inflammatory cytokine activation	Solid Tumors	1996 Phase I Completed
NCT00856336	Vadimezan (DMXAA/ASA404)	Pro-inflammatory cytokine activation	Refractory Tumors	2003 Phase I Completed
NCT00832494	Vadimezan (DMXAA/ASA404) Paclitaxel Carboplatin	Pro-inflammatory cytokine activation	NSCLC	2004 Phase I, II Completed
NCT00111618	Vadimezan (DMXAA/ASA404) Docetaxel	Pro-inflammatory cytokine activation	Hormone Refractory Metastatic Prostate Cancer	2005 Phase II Completed
NCT00630110	Vadimezan (DMXAA/ASA404) Docetaxel	Pro-inflammatory cytokine activation	Advanced or Recurrent Solid Tumors	2009 Phase I Completed
NCT01299415	Vadimezan (DMXAA/ASA404) Fluvoxamine	Pro-inflammatory cytokine activation	Solid Tumors	2009 Phase I Terminated
NCT01240642	Vadimezan (DMXAA/ASA404) Paclitaxel + Carboplatin Docetaxel	Pro-inflammatory cytokine activation	Metastatic Cancer With Impaired or Normal Renal Function	2010 Phase I Terminated
NCT01057342	Vadimezan (DMXAA/ASA404) Carboplatin Paclitaxel	Pro-inflammatory cytokine activation	Extensive-Stage Small-Cell Lung Cancer	2010 Phase II Completed
NCT00699517	Ombrabulin (AVE8062)	Tubulin	Advanced Soft Tissue Sarcoma	2008 Phase III Completed
NCT00968916	Ombrabulin (AVE8062)	Tubulin	Advanced Solid Tumors	2009 Phase I Completed
NCT01293630	Ombrabulin (AVE8062) Placlitaxel Carboplatin	Tubulin	Advanced Solid Tumors	2011 Phase I Completed
NCT01263886	Ombrabulin (AVE8062)	Tubulin	NSCLC Metastatic	2011 Phase II Completed
NCT01332656	Ombrabulin (AVE8062) Placlitaxel Carboplatin	Tubulin	Platinum-sensitive recurrent ovarian cancer	2011 Phase II Completed
NCT01034631	BNC105P Everolimus	Tubulin	Renal Cell Carcinoma	2010 Phase I, II Completed
NCT03454165	BNC105P Ibrutinib	Tubulin	Chronic Lymphocytic Leukemia	2018 Phase I Completed
NCT03647839	BNC 105P Nivolumab BBI608	Tubulin	Refractory Colorectal Cancer	2018 Phase II Completed
NCT00419328	NGR-hTNF	CD13 (aminopeptidase N)	Advanced Solid Tumors	2005 Phase I Completed
NCT00483080	NGR-hTNF	CD13 (aminopeptidase N)	Colorectal Cancer	2006 Phase II Completed
NCT00484211	NGR-hTNF	CD13 (aminopeptidase N)	Advanced or Metastatic Hepatocellular Carcinoma	2006 Phase II Completed
NCT00483093	NGR-hTNF Cisplatin	CD13 (aminopeptidase N)	Advanced or Metastatic Solid Tumor	2007 Phase I Completed
NCT00484276	NGR-hTNF	CD13 (aminopeptidase N)	Advanced or Metastatic Malignant Pleural Mesothelioma	2007 Phase II Completed
NCT00675012	NGR-hTNF Oxaliplatin Capecitabine	CD13 (aminopeptidase N)	Metastatic Colorectal Cancer	2007 Phase II Completed
NCT00484432	NGR-hTNF Doxorubicin	CD13 (aminopeptidase N)	Advanced or Metastatic Ovarian Cancer	2008 Phase II Completed
NCT00994097	NGR-hTNF Cisplatin Gemcitabine Pemetrexed	CD13 (aminopeptidase N)	Advanced NSCLC	2009 Phase II Completed
NCT00484341	Low-dose NGR-hTNF High-dose NGR-hTNF Doxorubicin	CD13 (aminopeptidase N)	Advanced Soft Tissue Sarcoma	2010 Phase II Completed
NCT03804866	NGR-hTNF Pegylated liposomal doxorubicin Doxorubicin	CD13 (aminopeptidase N)	Platinum-resistant Ovarian Cancer	2013 Phase II Completed

## Abbreviations

The following abbreviations are used in this manuscript:

AAT	Anti-angiogenic therapy
ASA404	Vadimezan
AVE8061	Ombrabulin
CA4P	Fosbretab
DMXAA	Vadimezan
EC	Endothelial cell
EMT	Epithelial-to-mesenchymal transition
EPCs	Endothelial progenitor cells
HIF-1α	Hypoxia-inducible factor 1α
HREs	Hypoxia response elements
LLC	Lewis lung carcinoma
LNPs	Nucleic acid-loaded nanoparticles
MHC	Major histocompatibility complex
NPI-2358	Plinabulin
NSCLC	Non-small cell lung cancer
PDAC	Pancreatic ductal adenocarcinoma
siRNA	Small interfering RNA
TAMs	Tumor-associated macrophages
TEMs	TIE2-expressing macrophages
TGF-β	Transforming growth factor β
TME	Tumor microenvironment
TNF-α	Tumor necrosis factor α
Treg	Regulatory T cells
VCAM-1	Vascular cell adhesion molecule 1
VEGF	Vascular endothelial growth factor
VEGFR2	Vascular endothelial growth factor receptor 2
VDAs	Vascular disruption agents
VCO	Vessel co-option
